# The Effect of Educational Video on COVID-19 and Dental Emergency Literacy among Students during Pandemic Era

**DOI:** 10.1055/s-0042-1743152

**Published:** 2022-04-18

**Authors:** Herry Novrinda, Risqa Rina Darwita, Khumaira Adienia Subagyo

**Affiliations:** 1 Department Dental Public Health and Preventive Dentistry, Faculty of Dentistry, Universitas Indonesia, Jakarta, Indonesia; 2 Faculty of Dentistry, Universitas Indonesia, Jakarta, Indonesia

**Keywords:** educational video, knowledge, COVID-19, dental emergency, indonesia

## Abstract

**Objectives** To determine (1) the level of knowledge regarding COVID-19 and dental emergency (DE) along with the factors that might be associated (2) with the effect of educational video on the level of knowledge among nondental students.

**Materials and Methods** This online pre-experimental study used an educational video regarding COVID-19 and DE with a one-group pretest–posttest design toward the undergraduate nondental students ( *n* = 363) from six public universities in West Java Province, Indonesia, aged 18 to 22 years. A Google Form was used and convenience sampling was undertaken.

**Statistical Analysis** The *chi-squared* , *Mann–Whitney* , *Wilcoxon* , and *Spearman correlation tests* were performed. *Cohen's d* *effect size* was used to assess the effect of videos.

**Results** Most of the respondents were females around 20.36 ± 0.94 years. There is a statistically significant difference between the pre- and posttest knowledge score for COVID-19 ( *p* <0.001) and DE ( *p* <0.001). The “with dental school” group had a significantly higher pretest score in DE literacy than the “without dental school” group. There is a significantly positive linear relationship between the frequency of watching videos and the posttest score. The effect size was 1.03 and 0.8 for COVID-19 and DE, respectively. The majority of students agreed (agree and very agree) with all statements regarding video education.

**Conclusions** The level of knowledge regarding COVID-19 and DE increased from 5.30 to 6.75 and 6.58 to 8.02, respectively. The associated factors were the type of university and the frequency of watching videos. Educational intervention seems to have a great effect in increasing the knowledge of nondental students.

## Introduction

The initial COVID-19 cases in Indonesia were detected in West Java Province (WJP), particularly Greater Jakarta, 1 which is the most populated province in Indonesia and one of the five provinces with the highest COVID-19 confirmed cases. 2 Since it is the most populated province in Indonesia, this area also has the highest number of dentists in Indonesia. The COVID-19 pandemic has an impact on all aspects of life including dental health services. The Indonesian Dental Association (PDGI) recommends dentists to postpone treatment without symptomatic complaints, elective, aesthetic treatment, and procedures using a drill/scaler/suction. 3 In addition, several organizations such as the Centers for Disease Control and Prevention (CDC) and the American Dental Association (ADA) recommend that dentists only prioritize emergency visits. 4 Therefore, dissemination of information and policy regarding COVID-19 and dental emergency (DE) is a necessity for the public in this pandemic era. Nowadays, it is common to use audiovisual technology for delivering information including video as an education media, 5 which makes the learning process more effective and understandable. In general, most of the students are familiar with new technologies to get latest information not only for educational purpose but also for their social life. A study in Jordan found that social media are also one of the main resources to get information regarding COVID-19. 6 Several previous studies have discussed COVID-19 literacy in various countries and communities 6 7 8 9 as well as emergency situations such as literacy in medical emergency situations among dental students 10 11 and knowledge of dental trauma or emergencies from nondental communities. 12 13 None of the above studies discussed COVID-19 and dental emergencies simultaneously with educational video interventions.

For our knowledge, there is a limited number (or none) of studies regarding knowledge of COVID-19 and DE during pandemic, especially among nondental students in Indonesia. Therefore, the aims of this study are to determine (1) the level of knowledge regarding COVID-19 and DE along with the factors that might be associated with (2) the effect of educational video on the level of knowledge among nondental students.

## Materials and Methods

This pre-experimental study used a one-group pretest–posttest design. The population used in this study were undergraduate students from six public universities in WJP. Dental students were excluded in this study. The total number of participants from the six universities was estimated at ∼80,000. The online *Raosoft* calculator was used to estimate the sample size. The power of test was set at 95% with a margin of error of 5% and a response distribution of 30% (assuming that nondental students might be less familiar with DE), resulting in a minimum sample size of 322 students. 14 This strategy had been performed in a previous similar study. 6 In order to anticipate the drop out during research process, we added participants so that a total of 401 students were selected with convenience sampling. In all, 363 students were fully analyzed.

The outcome variables were the level of knowledge regarding COVID-19 and DE, which were assessed before and after watching a 10-minute educational video (intervention variable).

A modified questionnaire from “ *Knowledge, attitudes, and practices towards COVID-19 among Chinese residents during the rapid rise period of the COVID-19 outbreak* ” 8 and *“Management of Acute Dental Problems during COVID-19 Pandemic* ” 15 was used to assess the students' level of knowledge. The first section of the questionnaire was designed to gather students' demographic information, which included the participant's name, phone number, gender, age, and the name of the university. The questionnaire on the level of knowledge for COVID-19 consisted of seven “Yes/No” questions about symptoms, treatment efforts, vulnerable groups, disease transmission, and prevention. The questionnaire on the level of knowledge for DE was made up of five “Yes/No” questions and four “multiple choice” questions about the different types of DE cases (situations that include or not include). For each question, if the answer was correct, then it will be scored 1 and if it was not correct, then it will be scored 0.

The questionnaire's validity and reliability were tested on 10% of the minimum total participants (40 students) who were not participants in this study. On each item of the knowledge questions, the correlation ( *r* ) was greater than 0.248 with a significance level ( *p* < 0.05). As a result, each of the questions was considerably valid. The reliability test yielded a score of 0.725 and was also considerably reliable.

This study period starts from August to October 2020 with the following stages: An informed consent form that included information on research procedures, objectives, and a statement of willingness to participate in this study was distributed to the students via a *Google Form* with *broadcast* . After that, the authors formed a *WhatsApp* group for participants who have completed a willingness statement. The *WhatsApp* numbers of participants were gathered by filling out the *Google Form* . In the next stage, the authors explained the research steps to the participants via *Zoom Meetings* in three sessions for three different student groups. The research steps were filling out the pretest questionnaire, watching educational video, and filling out the posttest questionnaire. A pretest was conducted via Google Form in which the link was shared in the WhatsApp group.

The educational video was given through Google Drive and the link was shared in WhatsApp private chat to the participants who had finished the pretest. The video contained information about COVID-19, which consisted of etiology, symptoms, mode of transmission, prevention, and incubation period for transmission and information about DE, which was “What Constitutes a Dental Emergency?” cases and emergency dental care by the ADA.

In addition to the questionnaire regarding COVID-19 and DE, a set of questions (consisted of four questions in Likert scale) was also given to the participants to obtain their opinion about the educational video. The participants were able to give their comments and suggestions regarding the video as well.

## Statistical Analysis

The Normality of data was tested with the *Kolmogorov–Smirnov* . A series of appropriate associative (such as chi-squared), comparative (Student's *t* -test or Mann–Whitney *U* test between groups and Wilcoxon Signed Rank Test for pretest–posttest within group), and correlative (such as Pearson or Spearman) were performed to assess the data. All the analyses were performed with SPSS version 23.0 (SPSS Inc., Chicago, Illinois, United States). *Cohen's d* e *ffect size* was used to assess the effect of the educational video in increasing the level of knowledge. 16

## Ethic Statement

The ethical approval for this study was obtained from the Faculty of Dentistry Universitas Indonesia Research Ethic Committee (approval no. 19/Ethical Approval/FKGUI/VIII/2020) and an electronic informed consent (via *Google Form* ) was obtained from each participant.

## Results

In all, 363 of 401 participants followed the research stages completely (response rate 90.97%). The majority of participants were females, age 20.36 ± 0.94 years and from University A (26.4%).

The female group showed an insignificantly higher score than males in the pretest–posttest COVID-19 and a significantly higher score than males in pretest DE as shown in Table 1 . The “with dental school” group had a significantly higher pretest score in DE literacy than the “without dental school” group.

**Table 1:** Score of knowledge regarding COVID-19 and DE among nondental students according to several variables

Variables		*n* (%)	COVID-19 score (¯ *X* ± SD)		DE score (¯ *X* ± SD)	
			Pretest	Posttest	Pretest	Posttest
	All participants	363 (100)	5.30 ± 1.09	6.75 ± 0.55 ^W^ **	6.58 ± 2.00	8.02 ± 1.34 ^W^ **
Gender	Male	77 (21.2)	5.23 ± 1.20	6.74 ± 0.52 ^W^ **	5.86 ± 2.25	8.08 ± 1.27 ^W^ **
	Female	286 (78.8)	5.32 ± 1.06	6.75 ± 0.56 ^W^ **	6.78 ± 1.89 ^M^ *	8.00 ± 1.36 ^W^ **
Age (y)	18–20	183 (50.4)	5.33 ± 1.03	6.77 ± 1.98 ^W^ **	6.49 ± 0.52	8.02 ± 1.31 ^W^ **
	21–22	180 (49.6)	5.27 ± 1.15	6.68 ± 2.02 ^W^ **	6.73 ± 0.59	8.02 ± 1.38 ^W^ **
Type of university	Without dental school	182 (50.1)	5.43 ± 1.11	6.28 ± 2.03 ^W^ **	6.68 ± 0.65	7.85 ± 1.41 ^W^ **
	With dental school	181 (49.9)	5.17 ± 1.06 ^M^ *	6.89 ± 1.93 ^W^ **	6.82 ± 0.43 ^M^ *	8.19 ± 1.25 ^W^ **
Frequency of watching video	>1	154 (42.4)	5.33 ± 1.06	7.10 ± 1.70 ^W^ **	6.94 ± 0.26	8.79 ± 0.49 ^W^ **
	1	209 (57.6)	5.28 ± 1.12	6.21 ± 2.13 ^W^ **/ ^M^ **	6.61 ± 0.66 ^M^ **	7.44 ± 1.48 ^W^ **/ ^M^ **

Note: W, obtained from Wilcoxon signed rank test; ** *p* < 0.001.

M, obtained from Mann–Whitney U test; * *p* < 0.05; ** *p* < 0.001.

There is a significant positive linear relationship ( *obtain from Spearman's correlation test* ) between the frequency of watching videos and the posttest scores of knowledge about COVID-19 ( *r* : 0.324; *p* < 0.001) and DE ( *r* : 0.603; *p* < 0.001), respectively. The effect size was 1.03 and 0.8 for COVID-19 and DE, respectively. It was categorized as large/big effect.

The majority of students agreed (agree and very agree) with all the statements as shown in Table 2 . For statement “ *I will watch the video again another time* ,” more than 30% were hesitant to agree, while for the statement “I want to apply what were suggested in the video,” 6.9% had no intention to apply.

**Table 2:** The opinion about the educational video among non-dental students

No.	Statements	Strongly disagree *n* (%)	Do not agree *n* (%)	Neutral *n* (%)	Agree *n* (%)	Strongly agree *n* (%)
1.	I found useful information on the video	2 (0.6)	0 (0)	10 (2.8)	91 (25.1)	260 (71.6)
2.	I learned new things about dental emergency	0 (0)	1 (0.3)	8 (2.2)	77 (21.2)	277 (76.3)
3.	I will watch the video again another time	9 (2.5)	24 (6.6)	94 (25.9)	134 (36.9)	102 (28.1)
4.	I want to apply what were suggested in the video	0 (0)	6 (1.7)	19 (5.2)	124 (34.2)	214 (59.0)

## Discussion

The students appeared to gain more knowledge about COVID-19 than about DE during the pretest stage. This could imply that students were more exposed to information about COVID-19 than about DE. This is understandable given that information about COVID-19 is widely disseminated in various media including social media 9 around the world. The insignificant difference in COVID-19 score between genders somehow is contrary to a study in Pakistan. 9 This difference might be from different characteristics. In this study, the participants were nondental students, while in the previous study in Pakistan the participants were dental students. On the other hand, students were still less familiar with DE. This finding supports the hypothesis that socialization or dissemination of DE is necessary during this pandemic. The university students from the dental school study program seemed to be more familiar with health issues especially COVID-19 and DE. Since the participants were nondental students, this finding might reveal the possibility of information exchange or transfer of knowledge/interest among students from this kind of university. The review on the students' prior knowledge would show the importance of educational intervention. This intervention showed that it might increase the level of knowledge among students, even from groups in which the level of prior knowledge was significantly different as also revealed in a study among undergraduate students in Brazil. 17 This study revealed issues regarding oral health literacy (OHL), in particular among nondental students. OHL is one of the agendas of various initiatives to improve oral health. 18 Thus, educational interventions, such as those conducted in this study, will be relevant in both the pandemic and new-normal eras.

The findings regarding correlation are consistent with the findings of Hamari et al, who observed that watching videos frequently can increase acquisition of knowledge. 19 The more often we interact with information in a learning process, the deeper our understanding of the information will be. This might be applicable not only to pedagogy but also to andragogy and heutagogy. Further research on the number of repetitions is required to achieve the optimal level of knowledge, which will become an opportunity in the future.

In terms of COVID-19, increase of knowledge after watching educational videos is in line with the findings regarding knowledge of COVID-19 on the Israeli population by Kaim et al. 20 In terms of OHL, this finding is also consistent with a conclusion in a systematic review from 13 studies that the level of knowledge can be increased through dental health education. 21 Since COVID-19 is still a relatively “new” disease, it takes more time to disseminate related information and implementation of several previously proven effective strategies is also worth trying. As demonstrated in this study, in the era of global pandemic, the use of electronic information media is one of the strategies that had increased knowledge significantly, particularly among nondental students in Indonesia. In higher education, video is now an important part of the information delivery process. 22 A meta-analyses conducted by Schmid et al have shown that videos increase knowledge. 23 Videos were used for biology because they can visualize many biological phenomena and are also thought to be interesting by students, according to a study by Stockwell et al. 24 Videos can also help increase knowledge and speed up the learning process. According to the findings of Latif et al in London, educational videos lasting more than 8 minutes can significantly improve participants' knowledge. 25

Although the effect size measurement seemed to have an issue with overestimation, several previous studies, including Kay, 21 Stockwell et al, 24 Lloyd and Robertson, 26 Hsin and Cigas, 27 showed that video was an effective educational tool. In addition, another study conducted by Kaim et al also reported a *Cohen's d effect size* 0.34. 20 These findings may support the idea that educational video interventions are still relevant and capable of improving (oral) health literacy during the COVID-19 pandemic. In this pandemic period, where many activities are done online, educational videos, both one-way and interactive videos, are certainly an alternative in conveying health messages or information. The large effect as found in this study is expected to motivate various parties to not worry about using educational videos.

The overall findings of this study should be weighed against some of its limitations. First, it should consider the limitations of online data collection, such as the inability to determine whether participants completed the questionnaire on their own or not, as well as the response rate. Second, despite the fact that the participants came from different universities, generalization of the findings may still necessitate additional research.

## Conclusions

The level of knowledge of COVID-19 and DE increased from 5.30 to 6.75 and 6.58 to 8.02, respectively. The associated factors were type of university and frequency of watching videos. Educational intervention seems to have a great effect in increasing the knowledge of nondental students. The effect size was 1.03 and 0.8 for COVID-19 and DE, respectively. Further research can be conducted by establishing a control group and an intervention group to determine the extent to which educational videos affect knowledge. A set of attitude questionnaires can be included to measure the improvement in participants' attitudes after viewing educational videos. To make each video shorter in length, the educational videos used can be divided into several videos.
